# Feeding critically ill patients at the right time of day

**DOI:** 10.1186/s13054-024-04994-0

**Published:** 2024-06-24

**Authors:** Hassan S. Dashti, YunZu Michele Wang, Melissa P. Knauert

**Affiliations:** 1 Department of Anesthesia, Critical Care and Pain Medicine, Massachusetts General Hospital and Harvard Medical School, 55 Fruit Street, Edwards 4-410C, Boston, MA 02114 USA; 2 Division of Bone Marrow Transplantation and Immune Deficiency, Cincinnati Children’s Hospital Medical Center, Cincinnati, OH USA; 3 Department of Pediatrics, University of Cincinnati College of Medicine, Cincinnati, OH USA; 4 Section of Pulmonary, Critical Care and Sleep Medicine, Department of Internal Medicine, Yale School of Medicine, New Haven, CT USA

Chrononutrition, a field of circadian medicine [1], examines the effects of the timing of eating on circadian rhythms, biological processes, and disease pathogenesis and treatment [2]. The circadian clock orchestrates 24-h endogenous cycles, termed circadian rhythms, that govern physiology and behavior [3]. Food intake is an environmental cue, similar though less potent than light exposure, that synchronizes human biology with the external environment. As a diurnal species, humans consume foods during the active phase (daytime); consuming foods during the inactive phase disrupts the system [4]. Key metabolic processes are blunted during the nighttime resulting in suboptimal nutrient metabolism due to circadian misalignment, a mismatch between the timing of eating and the circadian system [3]. Nutrient intake during the inactive phase may also reprogram the clocks of peripheral tissues and cause internal desynchronization between the impacted clocks and clocks in other tissues [5]. Chrononutrition is centered around aligning nutrition with the circadian system and is a research priority of the NIH [6].

Modern intensive care unit (ICU) environments and practices are known to be disruptive to circadian rhythms. Critically ill patients in the ICU are subjected to abnormal circadian entrainment signals including dim artificial lighting patterns and immobility [7]. It is also typical for critically ill patients to receive enteral or parenteral nutrition support in a continuous manner across all phases of the 24-h cycle [8]. The delivery of 24-h nutrition support is expected to exacerbate disruption. The historical practice of favoring a slower continuous rate has been widely adopted to limit the unsubstantiated universal risk of gastrointestinal intolerance and aspiration, along with consequent pneumonias, for enteral nutrition, and dysglycemia and other metabolic burden, for both feeding modalities [9]. Continuing nutrition support through the night is presumed to overcome missed calories when patients may have their feeds temporarily held during the daytime. Clinical guidelines make no guidance on the timing for nutrition support, and some suggest that the evidence for continuous feeding may be weak [8, 10]. Supporting the circadian health of critically ill patients through modern feeding schedules has the potential to improve metabolic outcomes by limiting circadian misalignment, and more broadly benefit robust circadian function, which is necessary for patient recovery.

Recent advancements in chronobiology, including studies on time-restricted eating [11], suggest that nutrition support should be cycled during the day in a time-restricted manner. Intermittent enteral feeding (providing feeds at 3 or 4 discreet times with a feeding pump during the day mimicking normal meals) and daytime cycles of parenteral nutrition (cycles that start and end during the day) are likely more physiologic. A recent pilot trial in pediatric hematopoietic stem cell transplant recipients showed that daytime infusion of parenteral nutrition is feasible and safe and is associated with a faster transition to an oral diet compared to 24-h continuous infusion [12]. Another pilot study showed that transitioning from overnight to daytime parenteral nutrition in patients with short bowel syndrome on home nutrition support is safe and not associated with dysglycemia [13]. Clinical trials in critically ill patients (including NCT04737200, NCT05551325, NCT05627167, NCT05795881, NCT04870554) will pave the way to the future of intensive care medicine. Expected benefits include providing adequate calories with limited interruptions to feeds, tighter glucose control, limited infection risk, maintained muscle mass, and improved sleep.

Daytime infusions of nutrition support may pose risks for some critically ill patients. Intermittent infusions of enteral nutrition require higher feeding rates ranging from 100 to 400 mL per hour. Some patients with elevated risk of aspiration due to severe gut dysmotility and structural abnormalities or those with postpyloric feeding tubes or peripheral intravenous access may not tolerate higher volumes; these populations may merit dedicated investigation regarding risks and benefits. Bolus feeding, a more rapid push method which delivers the meal in minutes using a catheter syringe, is not recommended for critically ill patients. In general, nutrition support should only be considered for patients with ICU stays of more than 48 h [10]. For patients initiating nutrition support, the proposed feeding regimen may be appropriate only during the chronic phase (anabolism) rather than the acute phase (catabolism) of illness. Another concern is augmented metabolic burden including dysglycemia associated with higher rates of infusions. Concern of dysglycemia is generally unfounded by empirical evidence. Recent pilot data suggests tighter daily glucose averages with daytime parenteral nutrition compared to 24-h continuous infusions [12]. It has also been hypothesized that overnight infusions may promote appetite. However, evidence suggests that daytime cycles of parenteral nutrition supports a faster transition to an oral diet [12]. Nevertheless, close monitoring of patient tolerance to daytime infusions including events of aspiration and dysglycemia, and adequate oral intake is necessary.

Daytime nutrition support is facilitated by recent scheduling features of electronic health records and automated infusion pumps; however, its implementation requires that hospital operations be reevaluated. Protocols on feeding tube placements and parenteral nutrition compounding may need to be recalibrated. Strategies for optimizing glycemia for patients with diabetes such as the administration of long-acting insulin need to be revised to account for discrete meals. Careful coordination by a multidisciplinary clinical team including nurses, dietitians, physicians, and pharmacists is necessary.

Continued innovation in nutrition support formula and technology will facilitate the drive towards an ICU supportive of circadian health. Whether shorter infusion cycles may elicit cardiometabolic benefits observed with time-restricted eating without augmenting metabolic burden is unknown [11]. The continued use of established mouse models for the study of nutrition support and chrononutrition will support this effort [2, 14]. Methods to personalize feeding schedules based on each patient’s biological timing rather than societal clock timing remain to be determined [15]. It is possible that daytime nutrition support concomitant with other effective interventions through a chrono-bundle encompassing light, food intake, and physical function (other synchronizers of the circadian system) may be more effective [7]. Existing non-pharmacologic ICU circadian interventions include upgrading light technology and utilizing solar shades.

In summary, the default clinical practice of 24-h continuous nutrition for critically ill patients should be reconsidered given its lack of evidence of safety and efficacy over more physiologic daytime feeds. Impending trials on the safety and efficacy of daytime feeds in critically ill patients are expected to inform timing considerations for nutrition support.

## Data Availability

Data sharing is not applicable to this article as no datasets were generated or analyzed during the current study.

## Abbreviation

ICU: Intensive care unit
